# Improving Surface Properties of PEEK for Dental Applications by Using Piranha Solution

**DOI:** 10.1155/2023/7840601

**Published:** 2023-09-20

**Authors:** Mohammed A. Alsmael, Aseel Mohammed Al-Khafaji

**Affiliations:** Department of Prosthodontics, College of Dentistry, University of Baghdad, Baghdad, Iraq

## Abstract

**Background:**

“Polyetheretherketone (PEEK)” is a biocompatible, high-strength polymer that is well-suited for use in dental applications due to its unique properties. However, achieving good adhesion between PEEK and hydrophilic materials such as dental adhesives or cement can be challenging. Also, this hydrophobicity may affect the use of PEEK as an implant material. Surface treatment or conditioning is often necessary to improve surface properties. The piranha solution is the treatment of choice to be explored for this purpose.

**Methods:**

PEEK disks of 10 mm diameter and 2 mm thickness were used in this study. Those samples were divided into five groups (each group has five samples). The first is the control group, in which no acid treatment was used; the second group undergoes sulfuric acid treatment. The remaining three groups were treated with Piranha solution; each group used a different concentration (1 : 3, 1 : 5, and 1 : 7 hydrogen peroxide to sulfuric acid, respectively). The period of treatment was 60 s for all groups. Wettability and surface roughness tests were done for the five groups. In statistical analysis, Shapiro–Wilk test was used to check the assumption of normality and to determine the statistical significance among groups; a one-way analysis of variance was employed. Subsequently, for multiple comparisons, Tukey's honestly significant difference post hoc test was performed.

**Results:**

The Piranha solution treatment groups showed a higher wettability compared to the control group and the group treated with sulfuric acid. Additionally, the Piranha solution treatment with a higher concentration of hydrogen peroxide (1 : 3) resulted in greater improvement in surface roughness compared to the control group and the lower concentration groups (1 : 5 and 1 : 7), while the sulfuric acid treated group showed the highest surface roughness.

**Conclusion:**

The results of this study suggest that the piranha solution can be an effective method for improving the surface characteristics of PEEK to be used in different dental applications, especially as a dental implant material, due to the increase in wettability and surface roughness.

## 1. Introduction

Polyetheretherketone (PEEK) is a biocompatible, high-strength polymer that has gained popularity in the field of dentistry. This material has a number of unique properties that make it well-suited for use in dental applications, including excellent wear resistance and strength, low coefficient of friction, and good biocompatibility [1, 2].

PEEK has been used in a variety of dental products, including crowns, bridges, and implant abutments [3]. In addition, PEEK has been explored as a material for use in dental implants, with research showing promise for its use as an alternative to traditional titanium implants [4].

One important aspect of using PEEK in dentistry is the need for proper surface treatment or surface conditioning. PEEK is a hydrophobic material, meaning it does not readily bond with water or other fluids. This can make it challenging to achieve good adhesion with dental adhesives or cement, which are typically hydrophilic materials [5].

To improve the bonding of PEEK with hydrophilic materials, it is often necessary to treat the surface of the PEEK with a conditioning agent or process [6]. This can involve using chemical or physical means to create a more porous, rough, or hydrophilic surface on the PEEK, which are the favorable surface characteristics of PEEK in dental applications [7]. Surface conditioning methods that have been explored for PEEK in dentistry include sandblasting, acid etching, and plasma treatment [8]. In the case of sandblasting or similar physical techniques, the objective is to augment surface roughness and increase surface area while maintaining the surface chemistry and polarity unchanged. However, chemical conditioning or acid etching diverges from this approach by simultaneously enhancing surface roughness and introducing varying degrees of chemical modification. It is important to carefully consider the surface treatment or conditioning of PEEK when using it in dental applications, as the bonding and long-term stability of the material may depend on it [9].

One method of surface treatment for PEEK that has been explored in dentistry is acid etching. Acid etching involves using an acidic solution to etch or roughen the surface of the PEEK, creating a more porous and hydrophilic surface. Sulfuric acid is one type of acid that has been used for this purpose [10].

Another method that has been used for surface conditioning of PEEK is the use of the Piranha solution. Piranha solution is a strong oxidizing agent that can be used to etch or clean the surface of PEEK [11]. Piranha solution provides enhanced surface activation of PEEK due to its strong oxidizing nature. This activation facilitates improved wettability, enabling better wetting and spreading of subsequent coatings or adhesives onto the PEEK surface. Consequently, this promotes stronger bonding and adhesion, enhancing the overall performance and durability of the material in various applications [12].

Furthermore, the Piranha solution offers a relatively fast and efficient treatment process for PEEK compared to alternative methods. Its aggressive nature allows for rapid and thorough cleaning and activation of the material, reducing processing time and improving workflow efficiency [13]. It is typically composed of a mixture of sulfuric acid and hydrogen peroxide and is highly effective at removing contaminants and creating a rough, hydrophilic surface on PEEK [14].

Both acid etching and the use of Piranha solution have been shown to improve the wettability and roughness of PEEK surfaces, which can enhance the bonding of PEEK with hydrophilic materials such as dental adhesives or cement [15].

It is important to carefully control the concentration and duration of the acid treatment, as excessive or prolonged exposure to acid can weaken PEEK's physical and mechanical properties. Also, it is important to handle the Piranha solution with caution, as it can be dangerous and should be used in a well-ventilated area with appropriate protective equipment [14]. As direct contact with piranha solution may cause chemical burns in the skin, also the inhalation of vapors could cause respiratory irritation or even permanent damage to the lungs. Also, due to its corrosive nature, it can cause significant damage to laboratory equipment, surfaces, and other materials [16].

Here in this study, the aim was to compare control PEEK samples (untreated) with PEEK samples treated with sulfuric acid and also with PEEK samples treated with Piranha solution in different concentrations (1 : 3, 1 : 5, and 1 : 7 hydrogen peroxide to sulfuric acid, respectively), the comparison was wettability and surface roughness. This study compared the treatment of PEEK with different concentrations of Piranha solution and compared wettability results with roughness measured by atomic force microscope (AFM), which was not done in any previous papers for these concentrations.

## 2. Materials and Methods

**Figure 1:**
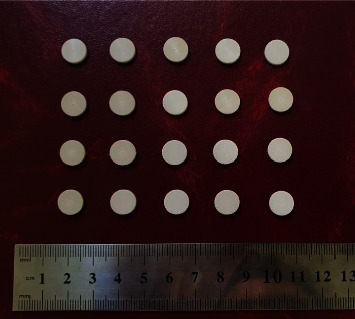
PEEK samples used in the study (round disks of 10 mm diameter and 2 mm thickness).

In this study, 10 mm round disks of PEEK were used with a thickness of 2 mm (Figure 1). These samples were obtained from a commercial supplier (Energetic Industry Co., Shenzhen, China). They were made by cutting extruded rods of PEEK. The samples were verified to be of consistent quality and dimensions and then smoothed by polishing with ascending order of sandpaper (500, 800, 1,200, 2,000, and 2,400 grit size). Also, sulfuric acid has a 98% concentration (Sulfuric Acid, Beckson Co., Connecticut, USA), and hydrogen peroxide has a 30% concentration (hydrogen peroxide 1.07209.1000, Emsure Co., Darmstadt, Germany). Piranha solution was prepared in different concentrations.

Five groups of PEEK samples were prepared (five samples for each), with each group containing five samples. The groups were as follows:

(1) Control group without acid treatment (C1).
(2) Treatment with sulfuric acid (98%) for 60 s (C2).
(3) Treatment with Piranha solution for 60 s,
  - Piranha solution (1 : 3 hydrogen peroxide to sulfuric acid) (P3).
  - Piranha solution (1 : 5 hydrogen peroxide to sulfuric acid) (P5).
  - Piranha solution (1 : 7 hydrogen peroxide to sulfuric acid) (P7).

The Piranha solution was prepared by mixing sulfuric acid and hydrogen peroxide in a specific ratio to achieve the desired concentrations. Preparation procedure was conducted in a specialized laboratory armed with specific equipment such as safety goggles, chemical-resistant gloves and apron, respiratory protective mask, face shield, and closed-toe shoes. The solutions were prepared in glass containers and were stirred until a homogeneous mixture was obtained. PEEK samples in the C2 group were submerged in a glass container of sulfuric acid for 60 s, while P3, P5, and P7 groups were submerged in piranha solution of the determined concentration (1 : 3, 1 : 5, and 1 : 7 hydrogen peroxide to sulfuric acid, respectively) also for 60 s [17, 18]. After removal of the samples from the glass container, they were cleaned ultrasonically in distilled water and in isopropyl alcohol each for 10 min, and then they were left to dry at room temperature for 15 min [19].

Finally, in order to select the most suitable surface treatment or concentration (which means increasing surface roughness and wettability), examinations of all control and experimental samples were done, roughness was assessed by AFM, and wettability was assessed by measuring the contact angle.

### 2.1. Roughness

To measure roughness, we used AFM (NaioAFM, Nanosurf, Basel, Switzerland). It was calibrated according to the manufacturer's instructions, and a 10 × 10 *µ*m scan size was used for all samples. Roughness values (*R*_a_) were calculated by the software of the device (Naio control software, v.3.10.0, Nanosurf, Basel, Switzerland) and reported in nanometers [20].

### 2.2. Wettability

To evaluate the wettability of PEEK samples, a contact angle goniometer (Ossila, Creating Nano Technologies Inc., Taipei, Taiwan) was used with a droplet of deionized water. The sample was horizontally positioned, and a droplet of 10 *μ*m of deionized water was released from a syringe (one drop on each sample). The droplet was allowed to disperse on the sample for 30 s, and then an image was taken. The contact angles were measured using computer software [21].

### 2.3. Statistical Analysis

Statistical analysis was conducted using the Statistical Package for the Social Sciences (SPSS v.27, IBM Co, New York, USA). The study results were presented using bar charts, where the mean values were indicated inside the bars, and the standard deviation was marked above the bars. Shapiro–Wilk test was used to determine the normal distribution of the results. To determine the statistical significance among groups, a one-way analysis of variance (ANOVA) was employed. Subsequently, for multiple comparisons, Tukey's HSD (honestly significant difference) post-hoc test was performed. A *P*-value greater than 0.05 was considered statistically nonsignificant (NS), while a *P*-value less than 0.05 was deemed statistically significant (S).

## 3. Results

### 3.1. Roughness

**Figure 2:**
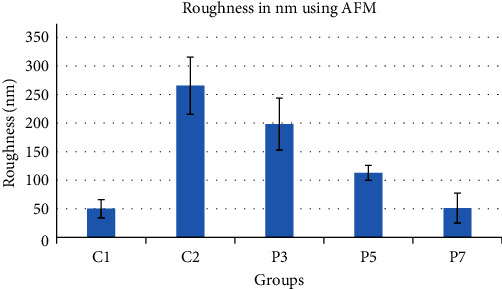
Bar chart showing average values and standard deviation of roughness values for control and experimental groups.

**Figure 3:**
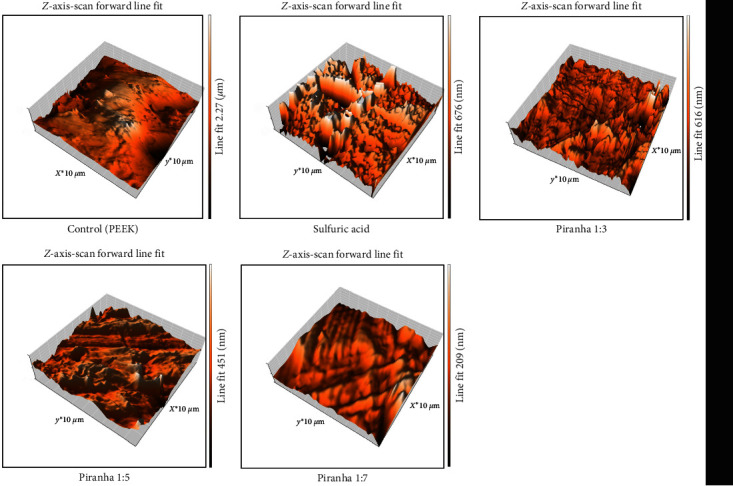
Surface topography of samples from different study groups was also obtained by AFM.

Roughness was assessed by comparing *R*_a_ values of the AFM test for different groups. The average roughness for the control group (C1) was the lowest (50.16 nm), and it was close to group (P7) with a reading of (51.257 nm), while sulfuric acid group (C2) showed the highest value (265.38 nm), regarding experimental groups they ranged between the two groups (C1 and C2) as shown in Figure 2 and Table 1. The surface topography of samples from different study groups is also shown in Figure 3. Topography shown in these pictures is consistent with the average readings of different groups.

Shapiro–Wilk test was used to assess the assumption of normality; it yielded *P*-values of 0.5941, 0.8226, 0.1874, 0.8642, and 0.4281 for the groups C1, C2, P3, P5, and P7, respectively, regarding these values it is not possible to reject the hypothesis of normality so it is assumed that the data is normally distributed for all groups (Table 2). Regarding descriptive statistics for these results, the *F* test of one-way ANOVA shows a highly significant difference in surface roughness (*P*-value = 0.000) among the five groups (C1, C2, P3, P5, and P7), as shown in Table 3.

For Tukey's multiple comparison tests, the results showed a significant difference between all groups except three pairs (C1 vs. P5, C1 vs. P7, and P5 vs. P7), as seen in Table 4.

### 3.2. Wettability

**Figure 4:**
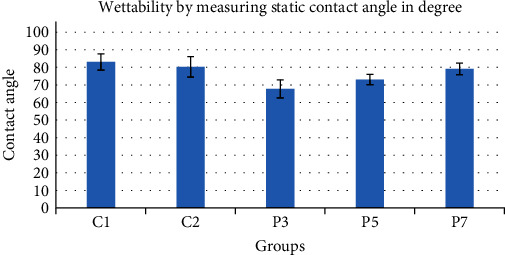
Bar chart showing average values and standard deviation of contact angle values for control and experimental groups.

Contact angle measurement was used to assess the wettability of the samples in the different groups. The mean value of the control group was 83.03°, which was the highest contact angle in all groups; the lowest value recorded was in P3 group with an average of 67.71°. Other groups (C2, P5, and P7) recorded a contact angle of (80.222, 73.09, and 79.07, respectively); results can be noted in Figure 4 and Table 5. It is worth noting that this method is a quantitative method as the reduction in contact angle means a higher wettability and vice versa.

Shapiro–Wilk test was used to assess the assumption of normality; it yielded *P*-values of 0.6786, 0.5474, 0.7641, 0.2526, and 0.9995 for the groups C1, C2, P3, P5, and P7, respectively, regarding these values it is not possible to reject the hypothesis of normality so it is assumed that the data is normally distributed for all groups (Table 2). The results indicate a highly significant difference in contact angle wettability among the five groups (C1, C2, P3, P5, and P7) according to the one-way ANOVA *F* test, as seen in Table 6.

Regarding Tukey's multiple comparison tests, four pairs showed a significant difference (C1 vs. P3, C1 vs. P5, C2 vs. P3, and P3 vs. P7), while all other six pairs showed NS differences (Table 7).

## 4. Discussion

The purpose of this research was to evaluate the effectiveness of surface treatment of PEEK with a mixture of sulfuric acid and hydrogen peroxide (piranha solution) in terms of improving the texture and surface qualities of a PEEK implant substrate. PEEK has established itself as a valuable material in implantology and other aspects of dentistry due to its biocompatibility, strong mechanical properties, and natural radiolucency. On the other hand, the bio-inertness of the PEEK surface and its hydrophobicity is a limitation to the material and to its wide use as implant material or as a major material in dental prosthesis [21].

In some surface modification techniques, sulfuric acid and other solutions containing it, such as piranha solution, are used to enhance the surface properties of PEEK material. For example, treatment of PEEK with sulfuric acid can create a rougher surface topography, which can improve the mechanical interlocking between the PEEK and some adhesives. This can be useful in applications such as bonding PEEK to metal or composite substrates [22].

The primary objective of this surface treatment is to balance between achieving a high surface roughness and obtaining a low contact angle. By achieving this balance, the bio-inertness of the material will be positively affected and become more suitable for adhering to other dental materials, thereby expanding its range of applications as a dental material in prosthodontics and as a substrate for dental implants [23]. The results of this study showed that the treatment of PEEK with sulfuric acid or piranha solution can improve the surface characteristics of the material.

An increase in the roughness means an increase in the surface area, which has a direct impact on the bonding and adhesiveness of PEEK to other materials, which permits a wider range of uses for PEEK as a dental material [24]. Piranha solution treatments resulted in lower roughness of PEEK compared with the C2 group, which may be attributed to the lower concentration of sulfuric acid and also to the consumption of sulfuric acid by reacting with hydrogen peroxide, which will reduce its ability in sulfonation of PEEK and making the oxidation of PEEK by hydrogen peroxide the main reaction that causes roughness of the surface. On the other hand, the increase in concentration of hydrogen peroxide among different groups of Piranha makes 1 : 3 concentration the highest in surface roughness because of the increase in oxidation of PEEK groups caused by hydrogen peroxide. This results in agree with dos Santos et al. [18].

Wettability is an important aspect of using PEEK in dentistry. As the turning of PEEK hydrophobicity into hydrophilicity means it can readily bond with water or other fluids like bonds or adhesives. Also, it increases cellular spreading and proliferation of cells on the surface of PEEK during implantation, which can make osseointegration faster and more reliable [17]. Piranha-treated groups showed increased wettability with increasing the hydrogen peroxide concentration, and this may be the effect of increased roughness, as stated by Wenzel's theory [25]. On the other hand, Piranha solution-treated PEEK samples showed a higher wettability compared with sulfuric acid-treated samples. This can be the result of the increased number of functional groups on the surface caused by the Piranha solution, as stated in multiple references [17, 18, 26]. The present report evaluated roughness and wettability. As previously done for composite dental materials and also for PEEK, future studies are needed to test other important characteristics, such as flexural strength [27], fatigue [28], roughness [29], and color stability [30], in order to complete the knowledge.

During surface treatment of PEEK, Piranha solution can cause extensive oxidation of the polymer surface, leading to great changes in its chemical and physical properties. If done in a controlled manner with the proper concentration and proper treatment time, then this surface treatment can result in great enhancement of the surface properties of PEEK [19]. In addition, during the use of piranha solution, when hydrogen peroxide reacts with sulfuric acid, the released oxygen reacts with the benzene group's aromatic ring of PEEK [12]. This causes the PEEK polymer to undergo oxidation, resulting in higher surface polarity and opening of the aromatic ring [31, 32]. This ultimately leads to an increase in the number of functional groups that can bind to surrounding tissues, so this might explain the decrease in the water contact angle of the samples that were treated with the piranha solution [33], especially for the P3 group as it was treated with the piranha solution containing a higher concentration of hydrogen peroxide compared to the P5, P7 groups and also compared with control group and the group C2 which was treated with a solution lacking hydrogen peroxide [34].

Regarding the relation between surface roughness and wettability, it is complex and depends on various factors, including surface chemistry and specific roughness characteristics [35]. In general and according to the Wenzel model, the wettability of the surface increases with an increase in surface roughness while holding other influencing factors constant. This can explain the increase in wettability among the Piranha-treated group. However, regarding the sulfuric acid treated group, the difference in surface chemistry may explain the reduction in surface wettability even with the increase in surface roughness [36].

Despite the positive results obtained from the surface treatment of PEEK with Piranha solution, there are several limitations to consider. The study focused solely on evaluating the effects of sulfuric acid and Piranha solution treatment on the surface characteristics of PEEK without investigating the long-term effects on the mechanical properties and biocompatibility of the material. Therefore, further research is needed to assess the durability and stability of the treated PEEK over an extended period, considering factors such as degradation, wear resistance, flexural strength, fatigue, color stability, and cytotoxicity in order to complete the knowledge.

## 5. Conclusion

Surface treatment with Piranha solution significantly improved the wettability and surface roughness of PEEK, with the most superior values observed were for Piranha solution with concentration of 1 : 3. However, it is suggested that acid treatment should be carefully controlled to avoid weakening the material, and Piranha solution or any other acidic solutions should be used with caution due to its potential hazards.

## Figures and Tables

**Table 1:** Data describing AFM results.

	Mean	Standard deviation	Minimum	Median	Maximum
C1	50.16308	16.0135728	32.77	44.781	69.827
C2	265.382	50.0911067	203.49	260.01	342.44
P3	198.274	45.43320515	148.17	207.87	241.56
P5	112.93	13.001971	93.25	111.66	127.39
P7	51.257	26.01263463	22.607	59.17	80.956

**Table 2:** Shapiro–Wilk test for all groups in AFM and wettability contact angle test.

Tests	Group C1		Group C2		Group P3		Group P5		Group P7	
	*P*-value	Passed	*P*-value	Passed	*P*-value	Passed	*P*-value	Passed	*P*-value	Passed
AFM	0.5941 NS	Yes	0.8226 NS	Yes	0.1874 NS	Yes	0.8642 NS	Yes	0.4281 NS	Yes

Wettability contact angle test	0.6786 NS	Yes	0.5474 NS	Yes	0.7641 NS	Yes	0.2526 NS	Yes	0.9995 NS	Yes

**Table 3:** One-way ANOVA test of roughness by AFM.

Test	Within groups			Between groups				
	Sum of squares	df	Mean square	Sum of squares	df	Mean square	*F*	Sig.
Roughness	22701.75	20	1135.088	178492.7	4	44623.17	39.313	0.000 (HS)

**Table 4:** Tukey's test of multiple comparisons for different groups of roughness test results.

Tukey's HSD test					
Groups pairs	Difference	Standard error	*Q* score	*P*-value	Sig.
C1 vs. C2	215.219	15.0671	14.2840	<0.00001	S
C1 vs. P3	148.111	15.0671	9.8301	0.00001	S
C1 vs. P5	62.7669	15.0671	4.1658	0.05499	NS
C1 vs. P7	1.0939	15.0671	0.0726	>0.99999	NS
C2 vs. P3	67.108	15.0671	4.4539	0.03612	S
C2 vs. P5	152.452	15.0671	10.1182	0.00001	S
C2 vs. P7	214.125	15.0671	14.2114	<0.00001	S
P3 vs. P5	85.344	15.0671	5.66423	0.00557	S
P3 vs. P7	147.017	15.0671	9.7575	0.00001	S
P5 vs. P7	61.673	15.0671	4.0932	0.06100	NS

**Table 5:** Data describing contact angle results.

	Mean	Standard deviation	Minimum	Median	Maximum
C1	83.032	4.575863	78.34	83.77	89.69
C2	80.222	5.846492	73.84	79.56	89.79
P3	67.714	5.056795	62.08	65.56	74.53
P5	73.09	2.925483	70.6	72.13	77.98
P7	79.07	3.271674	75.12	78.95	83.87

**Table 6:** One-way ANOVA test of wettability contact angle test.

Tests	Within groups			Between groups				
	Sum of squares	df	Mean square	Sum of squares	df	Mean square	*F*	Sig.
Water contact angle test	399.8139	20	19.99069	759.3411	4	189.8353	9.496	0.000 (HS)

**Table 7:** Tukey's test of multiple comparisons for different groups of wettability contact angle test.

Tukey's HSD test					
Groups pairs	Difference	Standard error	*Q* score	*P*-value	Sig.
C1 vs. C2	2.81	1.9995	1.4053	0.85506	NS
C1 vs. P3	15.318	1.9995	7.6608	0.00023	S
C1 vs. P5	9.942	1.9995	4.9721	0.01648	S
C1 vs. P7	3.962	1.9995	1.9815	0.63401	NS
C2 vs. P3	12.508	1.9995	6.2555	0.00217	S
C2 vs. P5	7.132	1.9995	3.5668	0.1252	NS
C2 vs. P7	1.152	1.9995	0.5761	0.99374	NS
P3 vs. P5	5.376	1.9995	2.6886	0.34858	NS
P3 vs. P7	11.356	1.9995	5.6793	0.00544	S
P5 vs. P7	5.98	1.9995	2.9907	0.2526	NS

## Data Availability

The data that support the findings of this study are available from the corresponding author upon reasonable request.
